# Deep learning ensemble models for CT-based differentiation of malignant and benign sacral bone tumors: development and evaluation

**DOI:** 10.1186/s13244-026-02220-9

**Published:** 2026-03-03

**Authors:** Ping Yin, Fei Zheng, Ke Liu, Kewei Liang, Li Yang, Lin Lu, Ning Lang, Yongmei Li, Nan Hong

**Affiliations:** 1 Department of Radiology, Peking University People’s Hospital, Beijing, P. R. China; 2 Department of Radiology, Peking University Third Hospital, Beijing, P. R. China; 3 Intelligent Manufacturing Research Institute, Visual 3D Medical Science and Technology Development, Beijing, P. R. China; 4 Department of Radiology, Shanxi Provincial People’s Hospital, Taiyuan, China; 5 Department of Radiology, Memorial Sloan-Kettering Cancer Center, New York, NY USA; 6 Department of Radiology, The First Affiliated Hospital of Chongqing Medical University, Chongqing, China

**Keywords:** Deep learning, Radiomics, Sacral tumors, Classification, Computed tomography

## Abstract

**Objective:**

Radiologists often face challenges in differentiating benign from malignant sacral bone lesions due to their similar imaging characteristics. This study aimed to develop an ensemble deep learning (DL) model that can preoperatively distinguish between benign and malignant sacral tumors using noncontrast computed tomography images.

**Materials and methods:**

Preoperative sacral CT scans from 569 patients with confirmed sacral lesions were analyzed. Data from Center 1 were utilized in model development and internal test via fivefold cross-validation, and those from Centers 2 and 3 were employed in external test. Various ensemble models combining human-readable interpretation and DL were developed. The diagnostic performance of the models and radiologists was assessed using metrics such as precision, recall, accuracy, area under the curve (AUC), F1 score, and confusion matrix. Furthermore, the clinical benefits derived from radiologists’ interpretations and supported by the DL model were evaluated.

**Results:**

The ensemble model, which integrates 3D-DenseNet121 with human interpretation, exhibited the most robust performance. The ensemble model demonstrated high performance on the internal and external test sets and achieved AUCs of 0.9139 and 0.8713, F1 scores of 0.9054 and 0.8571, precision of 0.9041 and 0.8824, recall of 0.9136 and 0.8333, and accuracy of 0.8630 and 0.8182, respectively. Across the external test cohort, all radiologists experienced improvements in AUC, accuracy, sensitivity, and specificity. Notably, junior radiologists demonstrated significant improvements compared with senior radiologists.

**Conclusion:**

The potential clinical application of the DL model lies in its capacity to considerably enhance the diagnostic efficiency of radiologists.

**Critical relevance statement:**

This study presents the first ensemble deep learning model integrating 3D-DenseNet121 with radiologists’ interpretation for preoperative differentiation of sacral tumors on noncontrast CT that improved diagnostic performance across all experience levels, particularly for junior radiologists.

**Key Points:**

- First artificial intelligence–radiologist ensemble for noncontrast computed tomography (NCCT)-based sacral tumor classification.
- Boosts all radiologists’ performance, with the greatest gains for juniors, potentially reducing referrals.
- Enables reliable NCCT diagnosis, overcoming contrast/magnetic resonance imaging dependency in musculoskeletal oncology.

**Graphical Abstract:**

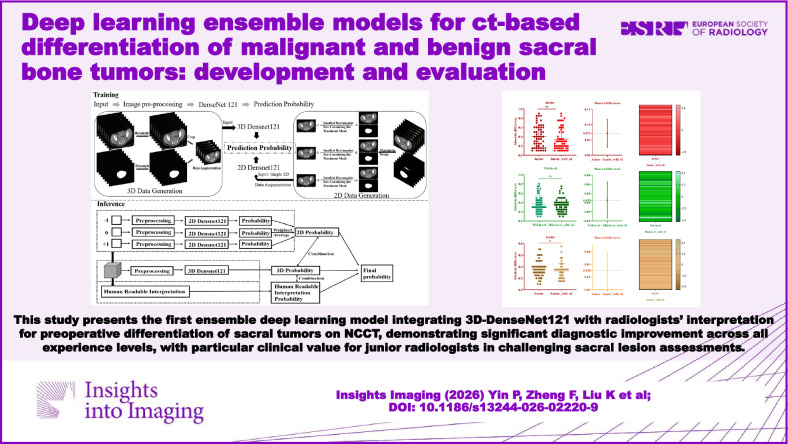

## Introduction

Bone tumors rank as the third leading cause of death among cancer patients under 20 years of age [1]. Sacral tumors can be classified into four types: primary malignant tumors (including sarcomas and chordomas), benign tumors, metastatic tumors, and tumors resulting from the local invasion of visceral malignancies [2]. Each type of bone tumor exhibits distinct biological behaviors, with benign tumors typically remaining stable. The sacrum provides a structural connection between the torso and lower half of the body and is subject to axial and rotational forces. Thus, tumors or their treatment can compromise the stability of the spinopelvic junction [2]. In the management of sacral tumors, less invasive procedures may be considered for benign variants to preserve neurological structures and maintain spinopelvic integrity. Conversely, treatment options for patients with malignant sacral tumors often involve chemotherapy, surgical resection, and radiotherapy [3–5]. Thus, accurate differential diagnosis plays a pivotal role in facilitating effective clinical decision-making in bone tumor cases.

Diagnosis of bone tumors relies on three main elements: symptomatology, imaging, and histopathological assessment [6]. However, the clinical presentation of sacral tumors is often nonspecific and typically characterized by neurological deficits and low back pain, which make early diagnosis challenging [7]. Although histopathological examination offers the most accurate diagnosis and guides treatment decisions, it represents the final step in the diagnostic process due to its invasive and time-consuming nature. Performing unnecessary biopsies on benign lesions incorrectly presumed malignant can lead to patient distress and postoperative complications, especially when conducted outside specialized multidisciplinary centers [8]. Moreover, certain contraindications, such as a platelet count below 50,000 or a bleeding tendency, may preclude biopsy [9]. In addition, biopsy procedures can yield nondiagnostic results in up to 30% of cases, which necessitates repeat procedures and increases the risk of complications [10].

Imaging plays a crucial role in the rapid differentiation of benign from malignant tumors. Various methods, including radiography, CT, MRI, angiography, scintigraphy, and positron emission tomography—CT, are used in bone tumor studies. CT imaging stands out for its high resolution and capability to detect small lesions (larger than 3 mm). It provides vital information on tumor matrix mineralization, calcification, ossification, cortical scalloping, destruction, and periosteal reaction, which aids radiologists in diagnosing bone tumors based on features such as location and margins [11]. However, given the low incidence rate of bone tumors, doctors lacking experience may misdiagnose them, which leads to unnecessary bone biopsies, patient discomfort, and increased costs, or they may miss a diagnosis altogether, which results in treatment delays or patient mortality [12]. In such cases, a sensitive and validated model for the characterization of suspicious bone lesions can reduce the rate of unnecessary referrals to higher levels of care and alleviate patient anxiety regarding potential cancer diagnoses. A computer-aided diagnostic tool capable of identifying benign lesions with a high specificity would be invaluable in reducing the rate of unnecessary biopsies by assisting radiologists in confidently ruling in malignancy [13].

Machine learning (ML) and deep learning (DL) approaches, which are data-driven, have garnered considerable attention in addressing complex medical challenges [14]. Convolutional neural networks, a subset of DL architectures, have exhibited remarkable performance across a range of computational tasks and have been adopted in medical imaging applications [15–18]. In addition, radiomics has been proven effective in distinguishing between benign and malignant lesions [19, 20]. However, research on DL applied to bone tumors remains limited due to the relatively low incidence rate of these tumors and the variability in their positions and pathology types, which results in challenges in the collection of sufficient imaging data [21]. Nonetheless, a few studies have demonstrated the potential of radiomics in distinguishing between benign and malignant sacral tumors [19, 20, 22]. This technique holds promise for improving the diagnosis and management of bone tumors.

Hence, our study aimed to develop an ensemble DL model that can distinguish between benign and malignant sacral tumors preoperatively. Integrating such a model into the clinical workflow can assist less experienced radiologists or physicians in improving the accuracy of their diagnoses and subsequently guide the appropriate management or referral of these patients [13].

## Materials and methods

**Fig. 1:**
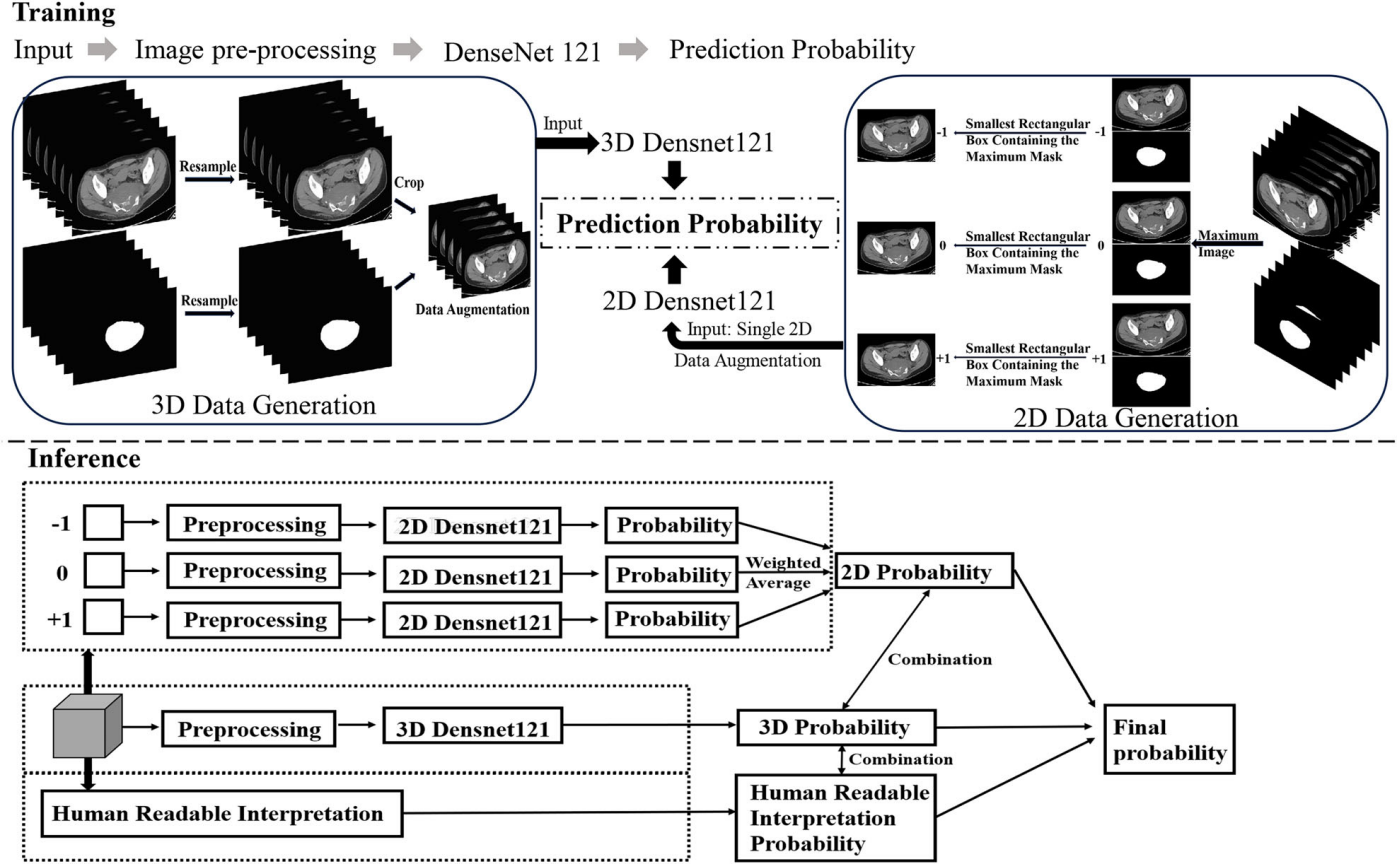
Overview of various ensemble models based on human-readable interpretation and DL. The ensemble model comprises two distinct phases: training and inference. In the training phase, two DL models were established, with 2D DenseNet121 and 3D DenseNet121 architectures utilized. During the inference stage, the predictive outputs from the 2D and 3D models were integrated with those from senior radiologists, and a weighted approach was applied to derive the final predictive outcomes of the ensemble models

To differentiate between benign and malignant sacral tumors preoperatively, different ensemble models based on human-readable interpretation and DL have been developed (Fig. 1). The ensemble model was developed through a two-phase process: training separate two-dimensional (2D) and 3D DenseNet121 models, followed by an inference phase that integrates their predictions with radiologists’ assessments through weighted fusion. The ensemble’s performance is ultimately compared against the interpretations of senior radiologists.

### Patient data and noncontrast computed tomography (NCCT) imaging

**Fig. 2:**
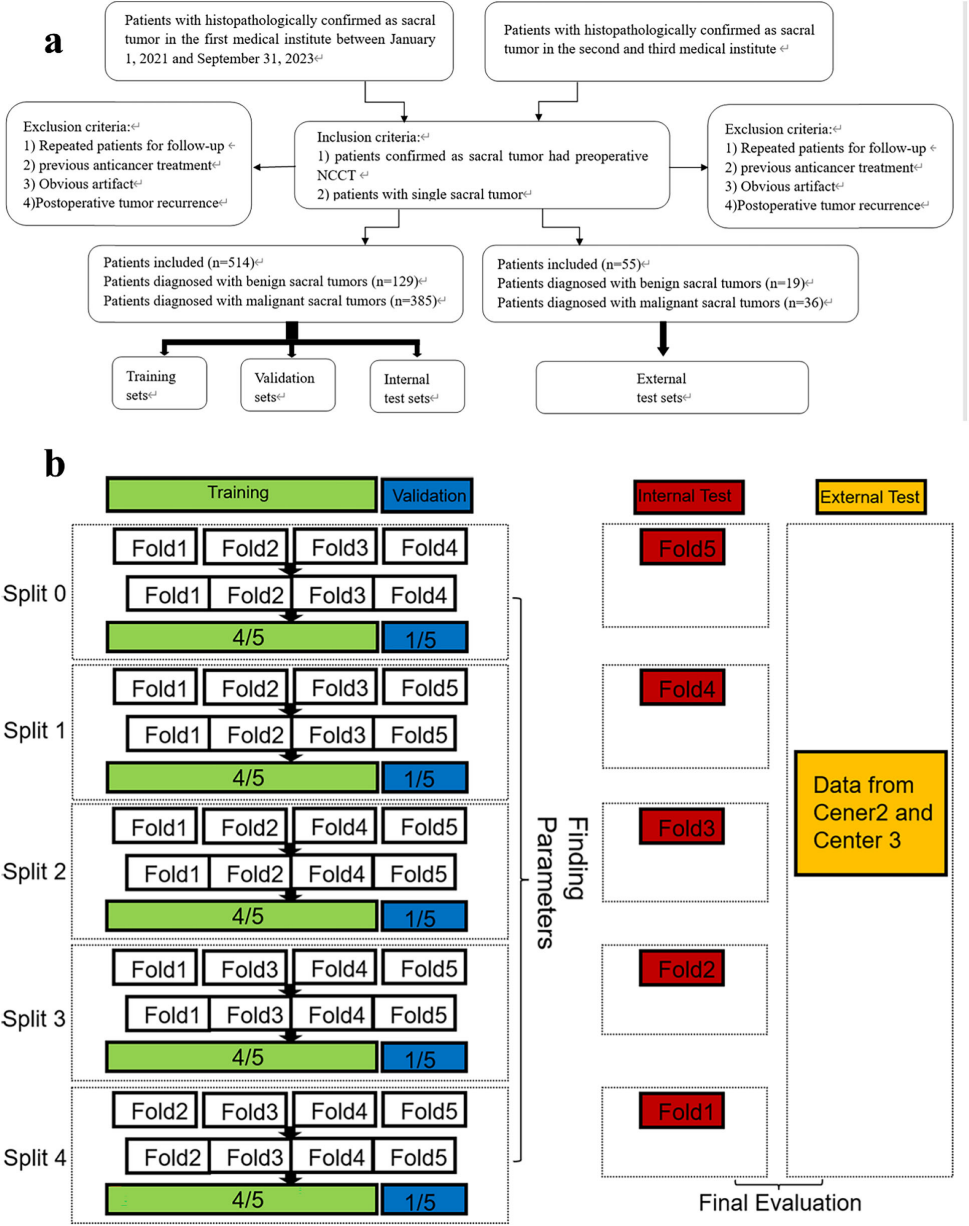
Patient selection workflow diagram (**a**) and schematic of fivefold cross-validation (**b**). Each subset sequentially served as the internal test set, and the remaining four subsets were utilized for model training and validation. This process involved training and evaluating the model across different subsets in each fold, which maximized data utilization and minimized the risk of overfitting

This retrospective study analyzed 569 patients with sacral tumors from three centers. A primary cohort (514 patients) was used for model development and internal validation via fivefold cross-validation and an external cohort (55 patients) for the final evaluation (Fig. 2). The observational study received approval from the Institutional Review Boards of Peking University People’s Hospital (No. 2024PHB568-001), with informed consent waived due to its retrospective design. All patients underwent preoperative sacral NCCT scans, with Digital Imaging and Communications in Medicine images converted to NiFTI format using dcm2niix software [23]; tumor regions of interest were manually segmented by an experienced radiologist. For comprehensive methodological details regarding patient demographics, NCCT image acquisition protocols, tumor segmentation procedures, and imaging parameters, please refer to the Supplementary Methods section.

### 3D and 2D DL analysis

Distinct preprocessing pipelines were implemented for the 3D and 2D DL analyses, with comprehensive methodological details provided in the Supplementary Methods section.

The preprocessed 3D images were analyzed using a 3D DenseNet121 model (https://github.com/ProjectMONAI/MONAI/blob/dev/monai/networks/nets/densenet.py), a DL architecture specifically designed for volumetric medical image analysis. The output of the fully connected layer was configured into two layers, which enabled the model to categorize patients into benign and malignant groups effectively. The transfer learning model utilized in this study was 2D DenseNet121, which was pretrained on the ImageNet dataset to initialize the weight values. Further detailed information regarding the DenseNet121 structure can be found at https://github.com/ProjectMONAI/MONAI/blob/dev/monai/networks/nets/densenet.py. Prior to training, the input 2D rectangular regions of interest were resized to dimensions of 224 × 224 pixel for DenseNet121.

The model was developed and evaluated using a fivefold cross-validation strategy on Cohort 1. In this scheme, the dataset was partitioned into five folds; for each fold, four folds were allocated for training/validation (split at an 8:2 ratio), and one fold was held out as the internal test set (Fig. 2b). This step ensured comprehensive utilization of the data for learning and unbiased assessment. All models were trained with the following unified configuration: a batch size of 20 and a cross-entropy loss function optimized by the Adam optimizer with a learning rate of 0.0001, for a total of 50 epochs. The training process involved standard forward and backward propagation to iteratively update the model parameters.

### Human-readable interpretation

Human-readable interpretation analysis was systematically conducted through two complementary evaluation phases: (1) baseline radiologist assessments and (2) model-assisted diagnostic evaluations.

In the first phase, an experienced senior radiologist established baseline diagnostic performance by evaluating all 569 lesions using comprehensive clinical data and imaging features. Assessments were scored on a rigorously defined 9-point confidence scale, with the 50% midpoint explicitly excluded to ensure definitive diagnostic categorization. These expert evaluations employed an identical fivefold cross-validation framework implemented for the DL model development, which enabled direct performance comparison between human and artificial intelligence (AI) assessments.

The second phase implemented a controlled evaluation of the DL model’s clinical utility through a multireader study design. Six radiologists representing three experience levels independently interpreted the external test cases in two separate reading sessions, with a mandatory 4-week interval implemented to mitigate potential recall bias. The initial session relied solely on conventional clinical and imaging data, and the subsequent session incorporated the 3D DenseNet121 predictions as decision support.

Complete methodological specifications are comprehensively documented in the Supplementary Methods section.

### Development and assessment of the human-DL fusion model

Following 2D and 3D DL analyses, the trained models—2D-DenseNet121 and 3D-DenseNet121—were developed. The application of fivefold cross-validation resulted in the generation of five 2D-DenseNet121 models and five 3D-DenseNet121 models. The internal and external test set data were fed into both models to generate prediction probabilities. Consequently, within the internal test set, the prediction probability of the 2D-DenseNet121 model represented the average prediction probabilities from the five 2D-DenseNet121 models, and the prediction probability of the 3D-DenseNet121 model was the average of the prediction probabilities from the five 3D-DenseNet121 models. Meanwhile, given that the 2D model processes three images per patient, the patient’s overall prediction probability is the weighted average of the three images’ probabilities. Image preprocessing mirrored that of the 2D and 3D DL analyses, which excluded data augmentation techniques such as random inversion.

Following the initial round of human-readable interpretation, probabilities derived from human interpretation were obtained. Subsequently, an ensemble learning methodology was employed to integrate multiple DL models or amalgamate these models with human-readable interpretation results. In the internal test set, the probability of the ensemble model was calculated as the weighted average of the predictions from the single DenseNet121 model and those made by a senior radiologist. In the external test set, the prediction results from the five DenseNet121 models were first averaged, and then, a weighted average of the average results and the predictions by senior radiologists was used to determine the prediction probability of the ensemble model. Specifically, the final prediction probability of the ensemble model was calculated as the weighted average of the probabilities derived from several configurations: the 2D-DenseNet121 combined with 3D-DenseNet121, the 3D-DenseNet121 combined with manual interpretation, and a combination of 2D and 3D-DenseNet121 with manual interpretation, as illustrated in the inference section of Fig. 1. The detailed formulas for these calculations are provided in Supplementary Materials.

### Statistical analysis

Differences in the clinical characteristics between the training and validation sets were evaluated using *t*-test and chi-square test. The analysis was conducted with statistical software SPSS 26 (SPSS Inc.), and statistical significance was defined as a *p*-value < 0.05. Model selection criteria were based on achieving the highest average area under the curve (AUC) score in the validation set. The model with the highest AUC in the validation set was selected for subsequent internal and external testing. This rigorous evaluation ensured the selection of the most robust model for subsequent analyses and applications. The model’s efficacy was assessed using the AUC and F1 score, which provided an overall measure of discrimination. Confusion matrices were utilized to quantify the model’s performance. Decision curve analysis (DCA) [24] and calibration curves [25] were utilized to determine the clinical utility of the ensemble models.

## Results

### Clinical characteristics

For Cohort 1, a total of 514 patients (277 men and 237 women) diagnosed with sacral tumors were enrolled. The mean age of the participants was 42.0 years, with an age distribution ranging from 6 years to 81 years. Cohorts 2 and 3 collectively included 55 patients with sacral tumors (27 men and 28 women). The mean age for these cohorts was 47.8 years, with participant ages spanning from 13 years to 82 years. Statistical analyses revealed significant differences in age and gender between the benign and malignant groups. Table 1 presents the detailed clinical characteristics of the patients.

**Table 1:** Baseline characteristics of the patients

	Cohort 1_ ALL	Cohort 1_ Benign	Cohort 1_ Malignant	*p*-value	Cohort 2&3_ ALL	Cohort 2&3_ Benign	Cohort 2&3_ Malignant	*p-*value
Age	42.05 ± 17.76	38.28 ± 13.51	43.31 ± 18.82	0.003	47.78 ± 16.46	43.95 ± 14.95	49.81 ± 17.06	0.213
Sex				0.028				0.109
Female	238 (46.30)	71 (55.04)	167 (43.38)		28 (50.91)	13 (68.42)	15 (41.67)	
Male	276 (53.70)	58 (44.96)	218 (56.62)		27 (49.09)	6 (31.58)	21 (58.33)	

### DL analysis and human-readable interpretation

**Fig. 3:**
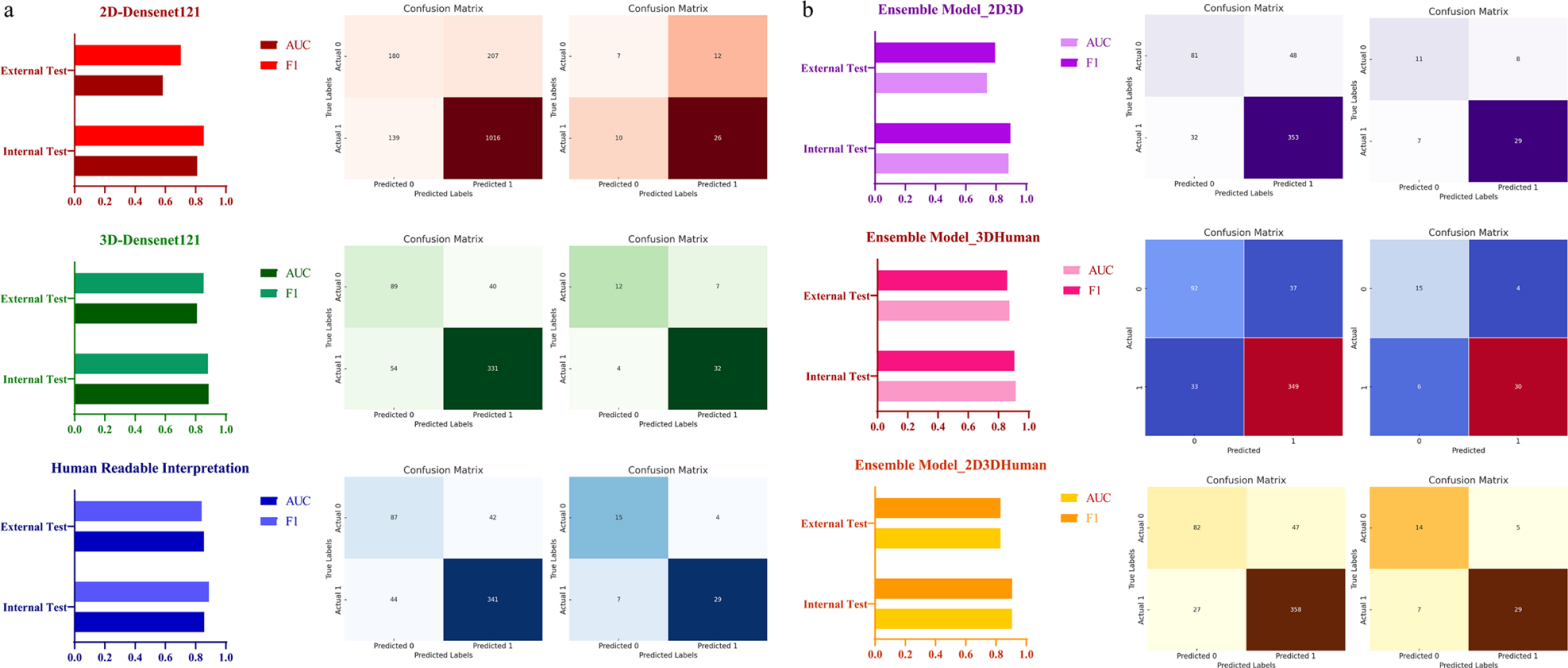
Model performance metrics across internal and external test sets, with the first column displaying AUC and F1 scores and columns 2 and 3 showing the corresponding confusion matrices for each model. **a** presents the results of a single deep learning model along with its human-readable interpretation, while **b** displays the results of the different ensemble models

The 3D DenseNet121 model demonstrated superior performance over the 2D DenseNet121 model in the internal and external test sets. It achieved an AUC of 0.8871 and an F1 score of 0.8823 in the internal test set, and an AUC of 0.8099 with an F1 score of 0.8533 in the external test set (Table 2 and Fig. 3a). The corresponding values for precision, recall, and accuracy are provided in Supplementary Table S1. Given the superior performance of the 3D DenseNet121 model, we have provided its attention heatmaps for representative examples of benign and malignant bone tumors (Supplementary Fig. 1). Furthermore, the attention patterns differed between benign and malignant tumors: in malignant cases, the heatmaps covered broader regions, which highlights invasive periosteal reactions and indistinct tumor boundaries and reflects their aggressive growth pattern. By contrast, for benign tumors, the heatmaps were generally more localized, focusing on the internal structure of the lesion. These visualizations enhanced the interpretability of the model by revealing distinct imaging features that drive its diagnostic decisions. Compared with the DL model, the interpretation of senior radiologists exhibited comparable performance in the internal test set, with an AUC of 0.8574 and an F1 score of 0.8879. In the external test set, senior interpretation outperformed the DL model, achieving an AUC of 0.8553 and an F1 score of 0.8406 (Table 2 and Fig. 3a). Table 2 and Fig. 3a also present the confusion matrices for the 3D DenseNet121 model, the 2D DenseNet121 model, and manual interpretation.

**Table 2:** AUC values, F1 scores, and confusion matrices of DL models and manual interpretation for differentiating benign and malignant sacral tumors

	Cross-validation split	AUC_ validation	F1-score_ validation	confusion matrix_ validation	AUC_ internal test	F1-score_ internal test	confusion matrix_ internal test	AUC_ external test	F1-score_ external test	confusion matrix_ external test
2D-Densenet121	0	0.8536	0.8743	[34 29]	0.7851	0.8428	[30 45]			
				[19 167]			[30 201]			
	1	0.8444	0.8642	[19 44]	0.8107	0.8649	[36 42]			
				[11 175]			[23 208]			
	2	0.8408	0.8871	[37 26]	0.7894	0.8505	[36 42]			
				[17 169]			[29 202]			
	3	0.7694	0.8351	[23 40]	0.8212	0.8344	[31 47]			
				[24 162]			[32 199]			
	4	0.7546	0.8108	[29 34]	0.8519	0.8803	[47 31]			
				[36 150]			[25 206]			
					**0.8112**	**0.8546**	**[180 207]**	0.5826	0.7027	[7 12]
							**[139 1016]**			[10 26]
3D-Densenet121	0	0.9439	0.885	[20 1]	0.8395	0.8378	[16 9]			
				[12 50]			[15 62]			
	1	0.8963	0.781	[14 9]	0.8686	0.8889	[18 8]			
				[6 56]			[9 68]			
	2	0.8909	0.8448	[16 5]	0.9091	0.8511	[22 4]			
				[13 49]			[17 60]			
	3	0.8372	0.8923	[11 10]	0.8881	0.8718	[15 11]			
				[4 58]			[9 68]			
	4	0.8349	0.8504	[10 11]	0.9301	0.9241	[18 8]			
				[8 54]			[4 73]			
					**0.8871**	**0.8823**	**[89 40]**	0.8099	0.8533	[12 7]
							**[54 331]**			[4 32]
Human-readable interpretation (senior)	0	0.8767	0.8926	[16 5]	0.8551	0.88	[18 7]			
				[8 54]			[11 66]			
	1	0.8664	0.878	[14 7]	0.7967	0.8846	[16 10]			
				[8 54]			[8 69]			
	2	0.8233	0.8618	[13 8]	0.8941	0.88	[19 7]			
				[9 53]			[11 66]			
	3	0.8157	0.8595	[14 7]	0.8824	0.9161	[19 7]			
				[10 52]			[6 71]			
	4	0.7769	0.8455	[12 9]	0.8589	0.879	[15 11]			
				[10 52]			[8 69]			
					**0.8574**	**0.8879**	**[87 42]**	0.8553	0.8406	[15 4]
							**[44 341]**			[7 29]

In the internal test set, the bold font indicates the average results derived from fivefold cross-validation. The confusion matrix is structured as [True Negatives (TN), False Positives (FP); False Negatives (FN), True Positives (TP)], where the top row corresponds to actual benign cases and the bottom row to actual malignant cases

**Fig. 4:**
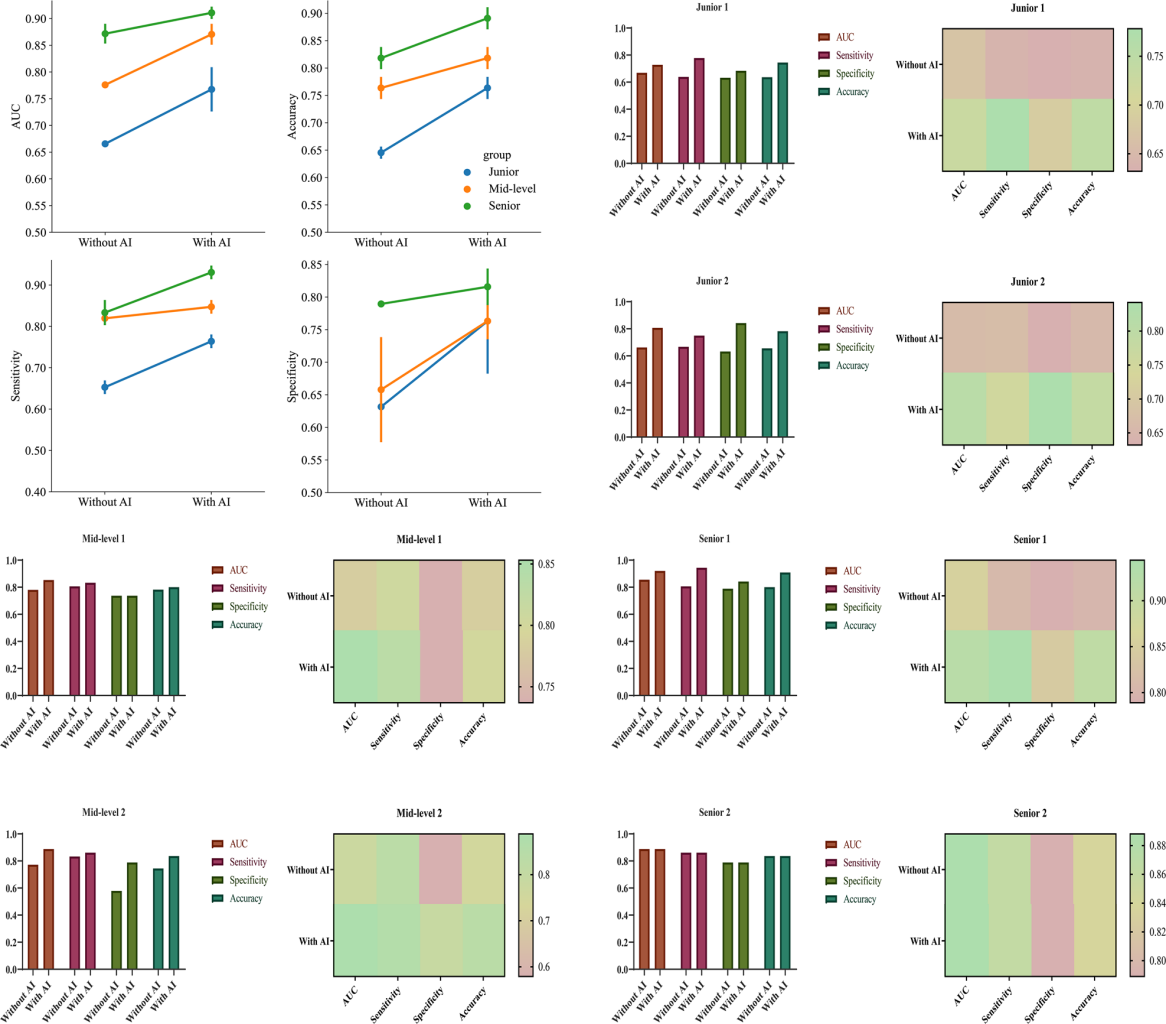
Comparison of the diagnostic performance (AUC, sensitivity, specificity, and accuracy) of three radiologist tiers (junior/mid-level/senior) with 3D DenseNet121 AI assistance versus those without. It also quantifies AI-induced improvements for six individual radiologists of different experience levels, visualized via performance improvement heatmaps

**Fig. 5:**
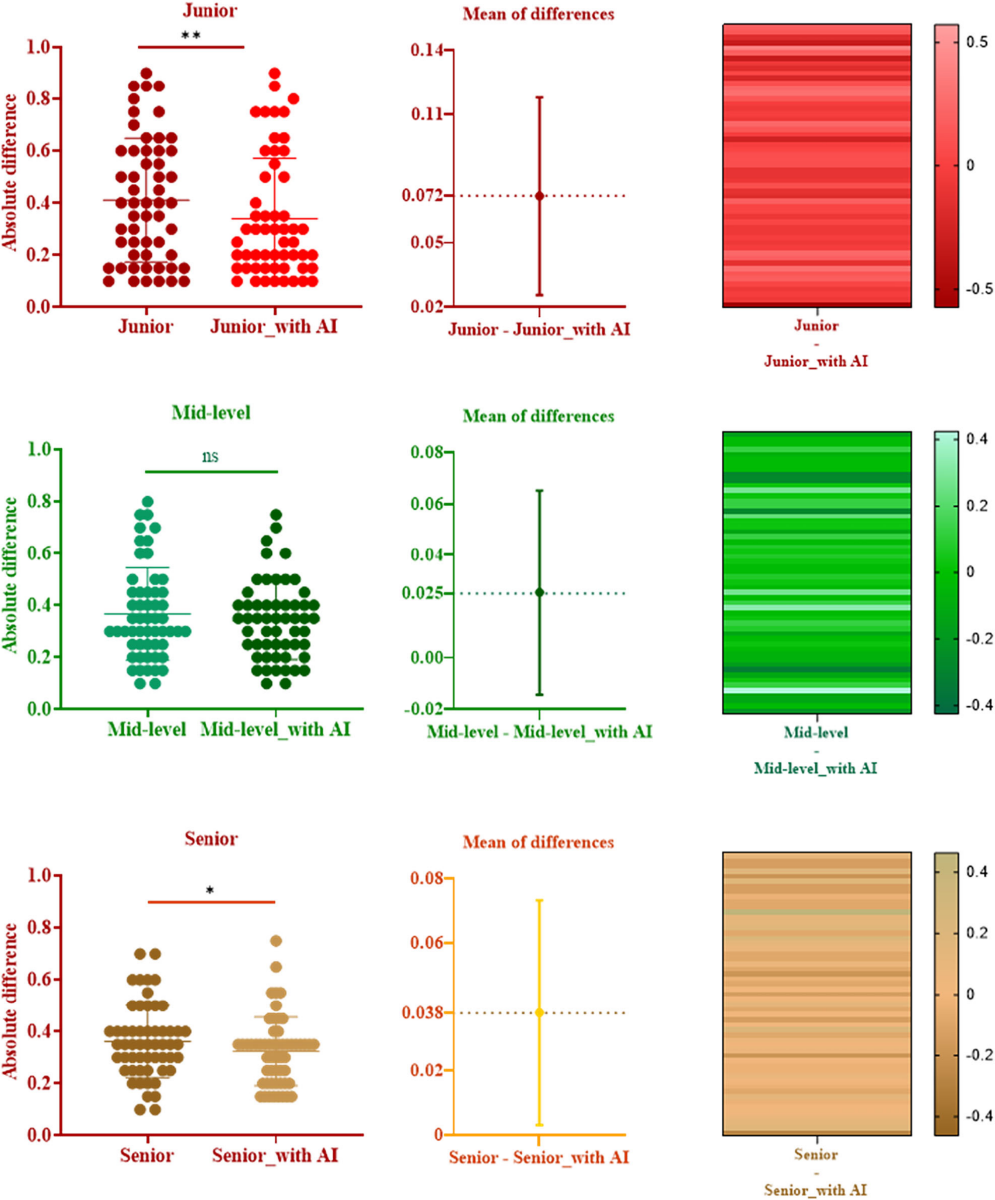
Key analyses: First column: absolute differences between radiologists’ diagnoses and ground truth values, which demonstrated an overall reduction in diagnostic discrepancies; Second column: improvement in these differences with AI assistance, which revealed that junior radiologists achieved the most substantial benefit (improvement of 0.072); Third column: heatmap visualization of diagnostic improvements for individual patients across all radiologists

The modifications in diagnostic outcomes made by six radiologists, both pre- and post-AI assistance, were meticulously examined in the second phase reader study (Table 3 and Supplementary Table S2). Figure 4 and Table 3 illustrate the alterations in decision-making, sensitivity, and specificity among junior, mid-level, and senior radiologists, with and without the aid of the 3D DenseNet121 AI model. In addition, we calculated the absolute difference between the radiologists’ interpretations and the true values to determine their diagnostic accuracy. A small difference indicates a high prediction accuracy. Figure 5 presents the absolute differences between radiologists’ interpretations and ground truth values, along with the improvement in these differences when aided by AI. A heatmap was used to further illustrate the diagnostic improvement for each individual patient. Within the external test cohort, all participating radiologists exhibited marked improvements in AUC, accuracy, sensitivity, and specificity metrics. Particularly, junior radiologists demonstrated significant enhancements in specificity when supported by AI. Moreover, the junior radiologists showed more pronounced improvements in predictive performance with AI assistance compared with their more experienced counterparts. Supplementary Fig. S1 provides the DeLong tests. Across the external test cohorts, the beneficial influence of the DL model in assisting radiologists to improve their overall diagnostic accuracy was clearly observable.

**Table 3:** Modifications in AUC values, sensitivity, specificity, precision, and accuracy for junior, mid-level, and senior radiologists enhanced by AI assistance in the external test set

Signature	AUC	Sensitivity	Specificity	Precision	Accuracy
Junior _Without AI	0.666	0.653	0.632	0.770	0.645
Mid-level _Without AI	0.776	0.819	0.658	0.821	0.764
Senior _Without AI	0.872	0.833	0.789	0.882	0.818
Junior _With AI	0.768	0.764	0.763	0.862	0.764
Mid-level _With AI	0.871	0.847	0.763	0.871	0.818
Senior _With AI	0.911	0.931	0.816	0.905	0.891

### Development and assessment of the ensemble model

Among the array of ensemble models evaluated, the combination of 3D-DenseNet121 with human interpretation exhibited the most robust performance. The ensemble model demonstrated a strong performance in the internal test set, with an AUC of 0.9139, an F1 score of 0.9054, and corresponding precision, recall, and accuracy of 0.9041, 0.9136, and 0.8630, respectively. Its performance in the external test set was robust, yielding an AUC of 0.8713, an F1 score of 0.8571, and precision, recall, and accuracy of 0.8824, 0.8333, and 0.8182, respectively (Supplementary Table S1). Table 4 and Fig. 3b depict the average confusion matrices for the ensemble model integrating 3D-DenseNet121 and manual interpretation, which highlight a notable improvement in the prediction accuracy for benign tumors.

**Table 4:** AUC values, F1 scores, and confusion matrices of the ensemble models for differentiating benign and malignant sacral tumors

	Cross validation split	AUC_ internal test	F1-score_ internal test	confusion matrix_ internal test	AUC_ external test	F1-score_ external test	confusion matrix_ external test
Ensemble model (3D-Densenet121 and 2D-Densenet121)	0	0.8499	0.8742	[17 8]			
				[11 66]			
	1	0.8691	0.8875	[14 12]			
				[6 71]			
	2	0.8946	0.9091	[19 7]			
				[7 70]			
	3	0.8701	0.8679	[13 13]			
				[6 71]			
	4	0.9251	0.9375	[18 8]			
				[2 75]			
		**0.8818**	**0.8952**	**[81 48]**	0.7405	0.7945	[11 8]
				**[32 353]**			[7 29]
Ensemble model (3D-Densenet121 and human-readable interpretation)	0	0.8821	0.8889	[17 8]			
				[6 68]			
	1	0.8821	0.8974	[17 9]			
				[7 70]			
	2	0.9461	0.9178	[24 2]			
				[10 67]			
	3	0.9176	0.8931	[15 11]			
				[6 71]			
	4	0.9416	0.9299	[19 7]			
				[4 73]			
		**0.9139**	**0.9054**	**[92 37]**	0.8713	0.8571	[15 4]
				**[33 349]**			[6 30]
Ensemble model (2D-Densenet121, 3D-Densenet121 and human-readable interpretation)	0	0.879	0.8974	[16 9]			
				[7 70]			
	1	0.8826	0.9068	[15 11]			
				[4 73]			
	2	0.9301	0.9032	[18 8]			
				[7 70]			
	3	0.8981	0.8861	[15 11]			
				[7 70]			
	4	0.9371	0.9375	[18 8]			
				[2 75]			
		**0.9054**	**0.9062**	**[82 47]**	0.8289	0.8286	[14 5]
				**[27 358]**			[7 29]

In the internal test set, the bold font indicates the average results derived from fivefold cross-validation. The confusion matrix is structured as [True Negatives (TN), False Positives (FP); False Negatives (FN), True Positives (TP)], where the top row corresponds to actual benign cases and the bottom row to actual malignant cases

**Fig. 6:**
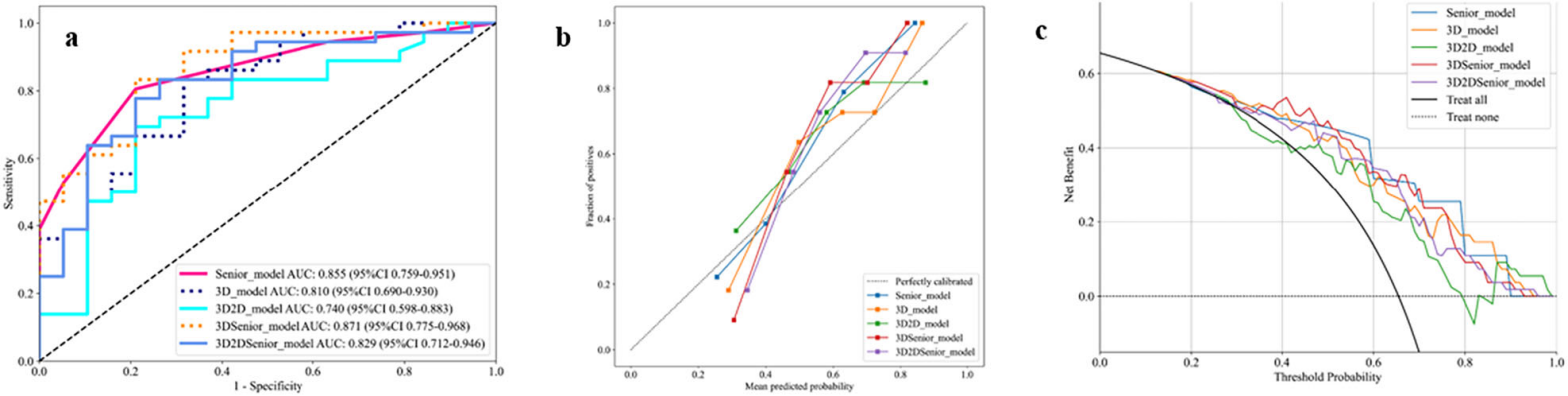
AUC (**a**), calibration curves (**b**), and DCA (**c**) for the senior radiologists. 3D DenseNet121 model, and ensemble models were plotted for the external test cohorts. Calibration curves were used to assess the concordance between predicted outcomes and observed events, and DCA was performed to quantify the net clinical benefit of the predictive models across various probability thresholds

Figure 6a displays all the models that demonstrated a strong performance. Figure 6 presents the calibration and DCA for the 3D-DenseNet121, manual interpretation, and all ensemble models. Figure 6b showcases a strong concordance between the predictions and actual observations in external test cohorts. DCA for all five models (Fig. 6c) indicated that each model offers a net clinical benefit over the “treat-all or none” strategy, with the ensemble models, particularly the one combining 3D-DenseNet121 and manual interpretation, outperforming the other classifiers. The differentiation between benign and malignant sacral tumors using DL models demonstrated superior clinical utility.

## Discussions

Our study expands the work of recent research that concentrated on predicting benign or malignant sacral tumors preoperatively [26–30]. Given the low prevalence of bone tumors, no external tests have been conducted across all published studies to date [26–29, 31]. This lack of external testing may lead to an overestimation of the predictive capabilities of radiomics. A prior random forest-based clinical-radiomics model reported a validation AUC of 0.899 for pelvic and sacral tumor classification [29]. In contrast to a study utilizing hand-crafted radiomics features, our work leverages an end-to-end DL approach. The use of this approach allowed our model to automatically learn discriminative features directly from CT images, moving beyond the limitations of predefined feature sets and contributing to its robust performance in clinical validation. Although Yin et al developed a deep neural network and ML classifiers, with their best clinical-LR model achieving an AUC of 0.84 [26], our work represents a paradigm shift. We introduced a human-AI collaborative ensemble model that significantly enhances radiologists’ diagnostic performance, which validates its practical utility as a clinical decision-support tool. Additional research has focused on differentiating specific types of sacral tumors [27, 28, 31]. Unlike subtype-specific models, our approach targets the foundational benign versus malignant decision, which is the most critical juncture in the diagnostic workflow and fundamentally guides all subsequent management. In our previous investigation, we evaluated various fusion radiomics approaches to differentiate benign from malignant sacral tumors [30]. The DL model demonstrated promising results, which prompted further exploration of its capabilities. This ongoing research aims to refine and expand the application of DL techniques in the accurate classification of sacral tumor pathologies.

Our findings also indicate that the performance of the 2D-based DL model was dissatisfactory and did not exhibit improvement in diagnostic accuracy when integrated with the results of the 3D DL model and senior radiologists. This observation aligns with clinical reality. The heterogeneous nature of bone tumor diseases results in a challenge in the comprehensive capturing of the characteristics of bone tumors based on a single image [32]. Meanwhile, the model’s performance was notably lower in the external test set compared with the internal test set. The diminished performance of the model in the external test set may be attributed to variance in data distribution and the test data falling outside the model’s learned scope [33, 34]. Notably, in Cohort 1, the number of malignant tumors was nearly thrice that of benign tumors, which potentially led to a deeper learning emphasis on malignant cases by the model. Conversely, in the external validation set comprising Cohorts 2 and 3, the ratio of malignant to benign tumors decreased to 1.9, which potentially elucidates the relatively diminished model performance observed. Furthermore, we compared for the first time the efficacy of DL models based on 2D and 3D images with that of human experts in discerning between benign and malignant sacral tumors. Our results indicate that DL models can effectively complement radiologists’ diagnostic processes. By integrating AI-generated insights into the clinical workflow, radiologists may benefit from reduced cognitive load, greater decision consistency, and increased diagnostic confidence. These advantages underscore the practical utility of incorporating DL models into routine practice [35–38].

A noteworthy aspect distinguishing this study from other radiomics investigations is the utilization of a two-round reader survey involving six radiologists. We investigated the practical advantages gained by radiologists derived from the assistance provided by DL in clinical practice. We posit that this exploration is particularly crucial given the anticipated auxiliary role of DL models in the future [39]. Despite the acknowledged strengths of AI and radiomics models, human experts will continue to wield ultimate decision-making authority. One primary rationale for this stance is the nascent state of interpretability concerning DL features, alongside the underexplored biological mechanisms underlying these radiomics features [40, 41]. Nonetheless, this consideration should not dissuade radiologists from leveraging radiomics methodologies to augment their diagnostic capabilities. Through the implementation of this assisting strategy during the second-round image interpretation, human experts demonstrated an overall improvement in AUC, accuracy specificity, and sensitivity for assessing benign and malignant sacral tumors. Although junior and senior radiologists derived positive assistance from the model, the former junior radiologists benefited to a greater extent. Consequently, this approach harbors the potential to expedite the learning curve of radiologists with limited experience [21, 35].

For the enhanced generalization ability and performance of ML models, two key strategies are employed: data augmentation [42] and fivefold cross-validation [43]. The study employed data augmentation (through rotation, translation, scaling, and flipping) to enhance dataset diversity and quantity. A rigorous fivefold cross-validation was implemented to optimize model robustness, mitigate overfitting, and ensure reliable performance evaluation. Crucially, cross-validation partitioning was performed at the patient level rather than at the image level to prevent data leakage, which guaranteed that all data from a single patient were confined to a single fold and ensured a truthful evaluation of model generalizability.

Our study had several limitations. First, despite being a multicenter study, the dataset was relatively small, particularly for the external validation cohort, which may constrain the statistical reliability and generalizability of the model. More importantly, the model’s performance was evaluated on a dataset with a significant imbalance, with a greater number of malignant sacral tumors collected compared with benign sacral tumors. Although this imbalance reflects the real-world incidence of these tumors [44], it inherently limits the assessment of generalizability, especially for benign cases. We therefore emphasize that future studies with larger, prospectively collected cohorts are essential to validate and refine the model. Second, this work is a retrospective study with cases identified from database searches, which might have introduced selection bias. In addition, given the retrospective nature of the study, we did not utilize multimodal sequences (CT and MRI), potentially affecting the efficacy of the DL strategy. Third, the demographic information, including age and sex, is also important and warrants exploration in future studies. Moreover, our study is inherently limited by its focus on binary classification (benign versus malignant). Although this approach is useful for initial triage, clinical decision-making critically depends on accurately distinguishing between specific tumor subtypes—such as chordoma, giant cell tumor, or Ewing sarcoma—as each requires a distinct management strategy and therapeutic pathway. Our model, in its current form, does not provide this essential subtype differentiation, which remains dependent on comprehensive histopathological and molecular evaluation. Future work should aim to develop multiclass classification models to address this significant clinical need. Finally, our dataset consisted exclusively of preoperative images and excluded any postoperative follow-up studies. After surgery, normal anatomical alterations, tissue repair responses, and potential complications can considerably alter the imaging landscape. These changes are further influenced by the tumor’s original location and the specific surgical technique employed, which collectively increase the complexity of image interpretation [45]. Our model’s posttreatment performance has not been evaluated and represents an essential direction for future research.

In summary, we have developed ensemble DL models that can accurately assess the malignancy of bone lesions preoperatively. Furthermore, we demonstrate that integrating the DL model enhances the radiologist’s decision-making process. This work underscores the potential of DL to assist radiologists in characterizing the malignancy of bone lesions with greater confidence and accuracy.

## ELECTRONIC SUPPLEMENTARY MATERIAL

Supplementary information

## Data Availability

The datasets generated or analyzed during the study are available from the corresponding author on reasonable request.

## Abbreviations

AUC: Area under the curve
DCA: Decision curve analysis
DL: Deep learning
ML: Machine learning
NCCT: Noncontrast computed tomography
