# Supplementary material for: Deep learning ensemble models for CT-based differentiation of malignant and benign sacral bone tumors: development and evaluation
Source: Insights Imaging. 2026 Mar 3;17:63. doi: 10.1186/s13244-026-02220-9 (PMC12957694; doi:10.1186/s13244-026-02220-9)
Supplement: Supplementary file 1 — Supplementary information [file 13244_2026_2220_MOESM1_ESM.pdf]

Deep Learning Ensemble Models for CT-Based Differentiation of Malignant and Benign Sacral Bone Tumors: Development and Evaluation

ELECTRONIC SUPPLEMENTARY MATERIAL

Patient Data

In this study, we gathered three independent patient cohorts from three centers. All patients presented with a single sacral tumor detected on NCCT within one month prior to their initial surgery. The primary cohort from Center 1 was utilized for model training, hyperparameter optimization, and internal testing, and was divided into a training set, a validation set, and an internal test set. This cohort comprised 514 patients from January 2011 to September 2023 (Figure 2a). Inclusion criteria encompassed histopathologically confirmed sacral tumors, with patients having preoperative NCCT images of single sacral tumors. Exclusion criteria included prior anticancer treatment, inadequate image quality, repeated patients for follow-up or monitoring, and postoperative tumor recurrence. Additionally, we gathered data for an external test cohort from Centers 2 and 3, specifically for the final model evaluation (Figure 2a). This cohort consisted of 55 patients with sacral tumors, and the inclusion and exclusion criteria remained consistent with those described earlier.

Furthermore, we implemented a 5-fold cross-validation scheme on the data from Cohort 1, wherein the dataset was partitioned into five subsets. Each subset sequentially served as the internal test set, with the remaining four subsets employed for model training and validation. During the training and validation phases, we randomly divided the eligible patients into a training set and validation set at an 8:2 ratio. This approach ensured that each segment of

the dataset was utilized as a training, validation, and internal testing set, thereby maximizing the use of the data (Figure 2b). Ultimately, the study encompassed a cohort of 569 patients diagnosed with sacral tumors, consisting of 303 males and 266 females. Additionally, the analysis included an assessment of the distribution of age and gender among the patients.

NCCT Images Acquisition and Tumor Segmentation

Raw NCCT data were acquired from the institute's Picture Archiving and Communication System in the Digital Imaging and Communications in Medicine (DICOM) format and subsequently transferred to a personal computer. DICOM images were then extracted and converted to the NiFTI format prior to analysis using the dcm2niix software. Manual outlining of the tumor region of interest (ROI) on the NCCT images was conducted layer by layer by a radiologist with 4 years of experience in skeletal muscle imaging, using ITK-SNAP 3.8.0 (http://www.itksnap.org). A second radiologist with 10 years of experience in musculoskeletal imaging was available for review and calibration.

The scanning parameters for the axial NCCT of each CT scanner.

All CT images were obtained using multi-detector row CT systems (Philips iCT 256; Philips Medical Systems, Best, Netherlands; GE Lightspeed VCT 64; GE Medical Systems, Chicago, IL, USA). The scanning parameters were as follows: 120 kV, 100-370 mAs, slice thickness = 5 mm, field of view = 350 mm × 350 mm, and matrix = 512 mm × 512 mm. The reconstruction methods were soft tissue and bone kernel algorithms.

The composition of benign and malignant sacral tumors included in each cohort of our study.

For the primary cohort at Center 1, the composition of the 129 benign tumors included 59 giant cell tumors (GCTs), 27 schwannomas, 23 neurofibromas, 6 solitary fibromas, 3 ependymomas, 3 hemangiomas, 3 chondroblastomas, 3 aneurysmal bone cysts, 1 bone cyst, and 1

paraganglioma. The 385 malignant tumors comprised 116 metastatic tumors, 84 chordomas, 75 osteosarcomas, 55 chondrosarcomas, 20 Ewing's sarcomas, 11 multiple myelomas, 8 malignant teratomas, 6 lymphomas, 5 liposarcomas, 2 undifferentiated sarcomas, 1 synovial sarcoma, 1 epithelioid sarcoma, and 1 malignant granulosa cell tumor.

In the external test cohort from Centers 2 and 3, the 19 benign tumors were categorized as 5 GCTs, 8 schwannomas, 2 neurofibromas, 2 hemangiomas, 1 angiolipoma, and 1 aneurysmal bone cyst. The 31 malignant tumors included 13 metastatic tumors, 12 chordomas, 2 Ewing's sarcomas, 1 osteosarcoma, 1 lymphoma, 1 liposarcoma, and 1 undifferentiated sarcoma.

3D and 2D deep learning preprocessing

3D deep learning preprocessing

Before being fed into the 3D training model, the image requires initial pre-processing, involving several essential steps. Firstly, the data is resampled to achieve voxel dimensions of [0.75, 0.75, 5]. Subsequently, the tumor region was cropped along its periphery to extract the 3D ROI containing the tumor. Following this, images input into the network are resized to [224, 224, 64]. Augmentation techniques are then deployed, encompassing a 50% probability of random rotation along the x-axis by 15 degrees, a 50% likelihood of horizontal flipping along the x-axis, and a 50% chance of zooming the image within a factor range of 0.9 to 1.1.

3D deep learning preprocessing

For 2D image acquisition, the process begins by selecting the axial slice within the smallest rectangular box containing the maximum mask, designated as the "maximum tumor image" in terms of NCCT images. Additionally, two additional images are extracted from the upper and lower slices adjacent to the maximum tumor image. In cases where the ROI is too small and the adjacent structure lacks three layers, the adjacent layer is utilized for filling. As a result, a total of 3 axial NCCT slices per patient are chosen and treated as individual samples for DL model development. It's ensured that three images based on the same patient can only appear simultaneously in the training set, validation set, or test data set. Data augmentation is carried out using the same methods as for 3D analysis.

Human Readable Interpretation

In the initial phase, a senior radiologist made preliminary diagnoses based solely on the patient's clinical data (demographics), raw NCCT images, and 3D ROI images to establish an initial diagnosis. A total of 569 lesions (514 lesions from Center 1 and 55 lesions from Centers 2 and 3) were presented in random order. Observers assessed all these tumor's category and its diagnostic classification on a scale ranging from 1 (signifying a confidence interval under 10%) to 9 (indicating a confidence interval between 90% to 100%). Lower scores on this scale suggest a higher probability of the tumor being benign, while higher scores indicate an increased likelihood of malignancy. It was mandated that a confidence level of 50% could not be chosen. To facilitate a comparative analysis of the DL model's performance against the diagnostic capabilities of senior radiologists in identifying benign and malignant sacral tumors, we also assessed the observations of senior radiologists using a "5-fold cross-validation approach", mirroring that of the DL model.

In a subsequent phase, an additional evaluation was conducted to compare the improved accuracy of the radiologists' tumor classifications with and without the DL model's assistance to investigate the clinical benefits derived from supporting the DL model. Six radiologists—two primary radiologists with three years of experience, two mid-level radiologists with five years, and two senior radiologists with over six years—interpreted the external test set images twice with a minimum four-week interval to reduce memory bias. For the second interpretation, they employed the 3D DenseNet121, which mimicked the initial review process to further analyze the benefits received from the model. Specifically, the radiologists integrated patient clinical data, original NCCT images, 3D ROI images, and 3D DenseNet121 predictions to make a refined diagnosis. Each radiologist independently assessed each external NCCT image twice—initially without, and subsequently with, the DL model's predictions—to differentiate between benign and malignant tumors. This paired evaluation design allowed for precise quantification of diagnostic accuracy improvements attributable to the AI assistance while controlling for confounding factors.

Development and Integration of the Human-DL Fusion Model

Following the 2D and 3D deep-learning analyses, we developed separate 2D-DenseNet121 and 3D-DenseNet121 models. Each architecture was trained using 5-fold cross-validation, resulting in five distinct models per architecture. For inference on the internal and external test sets, the prediction probability for each patient was generated as follows:

For the 2D-DenseNet121 model, each patient was represented by three images. The overall 2D prediction probability was computed as the average of the probabilities from these three images, which were themselves averaged across the five cross-validated 2D models.

For the 3D-DenseNet121 model, the prediction probability for each patient was obtained by averaging the outputs of the five cross-validated 3D models.

Subsequently, we constructed a hybrid DL-Radiologist fusion model using a weighted averaging strategy to integrate the predictions from the DL models and the radiologists' assessments. The final prediction probability of the ensemble model was calculated as the arithmetic mean of the included components, as defined below:

2D + 3D Fusion:

Final Score = (2D_Probability + 3D_Probability) / 2

3D + Radiologist Fusion:

Final Score = (3D_Probability + Radiologist_Score) / 2

2D + 3D + Radiologist Fusion:

Final Score = (2D_Probability + 3D_Probability + Radiologist_Score) / 3

Here, the Radiologist_Score was derived from the original observer ratings, which were recorded on a 9-point scale (excluding the middle score of 5). Scores of 1 to 4 were converted to probability values of 0.1 to 0.4, representing increasing confidence in a benign classification. Conversely, scores of 6 to 9 were converted to probability values of 0.6 to 0.9, representing increasing confidence in a malignant classification. This weighted averaging scheme was applied consistently across both the internal and external test sets.

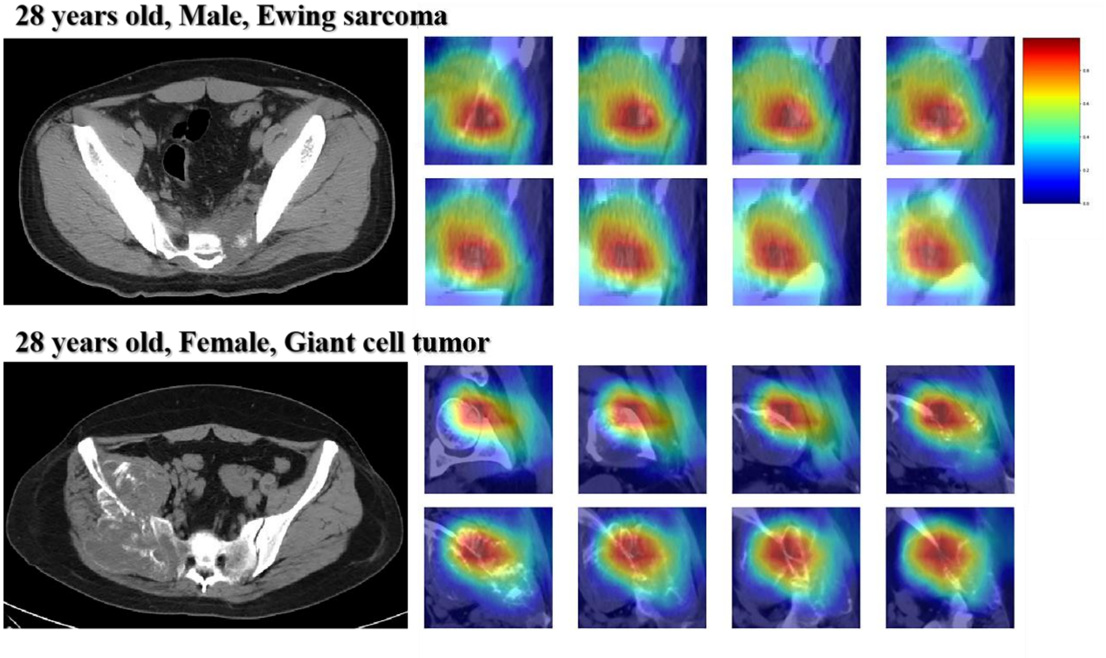

Supplementary Figure 1: displays representative examples of benign and malignant bone tumors—specifically, a giant cell tumor and a Ewing sarcoma, respectively. For each case, the original CT image is presented together with its corresponding attention heatmap. It should be noted that the heatmap is derived from an image that has undergone data augmentation, which included a 50% probability of random rotation by 15 degrees along the x-axis, a 50% chance of horizontal flipping along the x-axis, and a 50% possibility of zooming the image by a factor between 0.9 and 1.1. As a result, the spatial alignment of the heatmap may differ from that of the original CT image. The heatmaps are accompanied by a color scale (ranging from blue to red), where red indicates the regions that contributed most strongly to the model's classification decision.

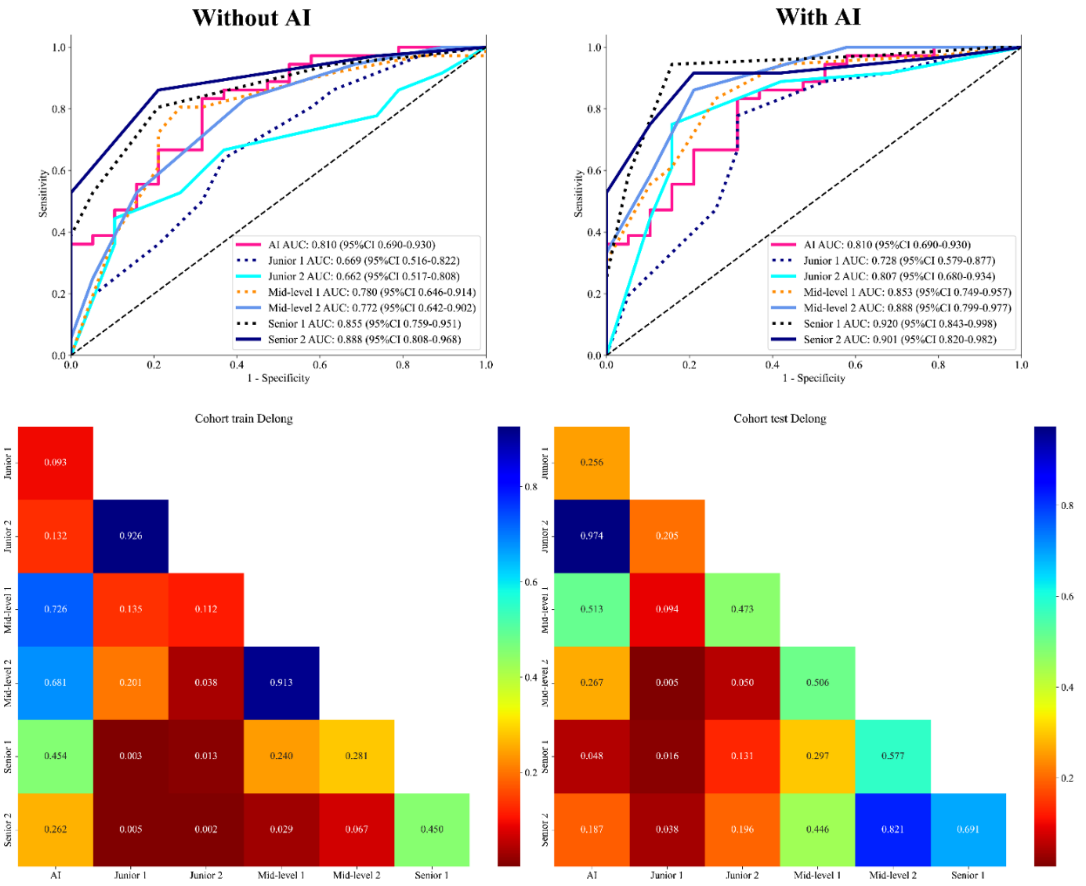

Supplementary Figure 2: AUC values and DeLong test results for human-readable interpretations with and without the assistance of 3D-DenseNet121 in an external test cohort.

Supplementary Table S1: Precision, recall, and accuracy of all models for differentiating benign and malignant sacral tumors.

	Cross validation Split	Precision_ Validation	Recall_ Validation	Accuracy_ Validation	Precision_ Internal Test	Recall_ Internal Test	Accuracy_ Internal Test	Precision_ External Test	Recall_ External Test	Accuracy_ External Test
2D-Densenet121	0	0.8520	0.8978	0.8072	0.8171	0.8701	0.7549			
	1	0.7991	0.9409	0.7791	0.8320	0.9004	0.7896			
	2	0.8667	0.9086	0.8273	0.8279	0.8745	0.7702			
	3	0.8020	0.8710	0.7430	0.8089	0.8615	0.7443			
	4	0.8152	0.8065	0.7189	0.8692	0.8918	0.8188			
					**0.8307**	**0.8797**	**0.7756**	0.6842	0.7222	0.6000
3D-Densenet121	0	0.9804	0.8065	0.8434	0.8732	0.8052	0.7647			
	1	0.8615	0.9032	0.8235	0.8947	0.8831	0.8350			
	2	0.9074	0.7903	0.7831	0.9375	0.7792	0.7961			
	3	0.8529	0.9355	0.8313	0.8608	0.8831	0.8058			
	4	0.8438	0.8710	0.7831	0.9012	0.9481	0.8835			
					**0.8922**	**0.8597**	**0.8171**	0.8205	0.8889	0.8000
Human Readable Interpretation (Senior)	0	0.9153	0.8710	0.8434	0.9041	0.8571	0.8235			
	1	0.8852	0.8710	0.8193	0.8734	0.8961	0.8252			
	2	0.8689	0.8548	0.7952	0.9041	0.8571	0.8252			
	3	0.8814	0.8387	0.7952	0.9103	0.9221	0.8738			
	4	0.8525	0.8387	0.7711	0.8625	0.8961	0.8155			
					**0.8903**	**0.8857**	**0.8327**	0.8788	0.8056	0.8000

Ensemble	0	0.8919	0.8571	0.8137			
Model	1	0.8554	0.9221	0.8252			
(3D and 2D-	2	0.9091	0.9091	0.8641			
Densenet121)	3	0.8452	0.9221	0.8155			
	4	0.9036	0.9740	0.9029			
		**0.8803**	**0.9169**	**0.8444**	0.7838	0.8056	0.7273
Ensemble	0	0.8947	0.9189	0.8586			
Model	1	0.8861	0.9091	0.8447			
(3D-	2	0.9710	0.8701	0.8835			
Densenet121	3	0.8659	0.9221	0.8350			
and Human	4	0.9125	0.9481	0.8932			
Readable							
Interpretation)		**0.9041**	**0.9136**	**0.8630**	0.8824	0.8333	0.8182
Ensemble	0	0.8861	0.9091	0.8431			
Model	1	0.8690	0.9481	0.8544			
(2D, 3D-	2	0.8974	0.9091	0.8544			
Densenet121	3	0.8642	0.9091	0.8252			
and Human	4	0.9036	0.9740	0.9029			
Readable							
Interpretation)		**0.8840**	**0.9299**	**0.8560**	0.8529	0.8056	0.7818

Note: In the internal test set, the bold font indicates the average results derived from five-fold cross-validation.

Supplementary Table S2: The modifications in AUC values, sensitivity, specificity, precision and accuracy for junior, mid-level and senior radiologists enhanced by AI assistance in the external test set.

Signature	AUC	Sensitivity	Specificity	Precision	Accuracy
Junior 1_Without AI	0.669	0.639	0.632	0.767	0.636
Junior 2_Without AI	0.662	0.667	0.632	0.774	0.655
Mid-level 1_Without AI	0.780	0.806	0.737	0.853	0.782
Mid-level 2_Without AI	0.772	0.833	0.579	0.789	0.745
Senior 1_Without AI	0.855	0.806	0.789	0.879	0.800
Senior 2_Without AI	0.888	0.861	0.789	0.886	0.836
Junior 1_With AI	0.728	0.778	0.684	0.824	0.745
Junior 2_With AI	0.807	0.750	0.842	0.900	0.782
Mid-level 1_With AI	0.853	0.833	0.737	0.857	0.800
Mid-level 2_With AI	0.888	0.861	0.789	0.886	0.836
Senior 1_With AI	0.920	0.944	0.842	0.919	0.909
Senior 2_With AI	0.901	0.917	0.789	0.892	0.873
